# Synthesis and Characterization of Ni-Pt Alloy Thin Films Prepared by Supercritical Fluid Chemical Deposition Technique

**DOI:** 10.3390/nano11010151

**Published:** 2021-01-09

**Authors:** Sudiyarmanto, Eiichi Kondoh

**Affiliations:** 1 Integrated Graduate School of Medicine, Engineering, and Agricultural Sciences, University of Yamanashi, Kofu 400-8511, Japan; g18dtea3@yamanashi.ac.jp or; 2 Research Center for Chemistry, Indonesian Institute of Sciences (LIPI), Kawasan PUSPIPTEK Serpong, Tangerang Selatan, Banten 15310, Indonesia

**Keywords:** supercritical fluids, deposition, Ni-Pt alloy films, thin films

## Abstract

Ni-Pt alloy thin films have been successfully synthesized and characterized; the films were prepared by the supercritical fluid chemical deposition (SFCD) technique from Ni(hfac)_2_**·**3H_2_O and Pt(hfac)_2_ precursors by hydrogen reduction. The results indicated that the deposition rate of the Ni-Pt alloy thin films decreased with increasing Ni content and gradually increased as the precursor concentration was increased. The film peaks determined by X-ray diffraction shifted to lower diffraction angles with decreasing Ni content. The deposited films were single-phase polycrystalline Ni-Pt solid solution and it exhibited smooth, continuous, and uniform distribution on the substrate for all elemental compositions as determined by scanning electron microscopy and scanning transmission electron microscopy analyses. In the X-ray photoelectron spectroscopy (XPS) analysis, the intensity of the Pt 4f peaks of the films decreased as the Ni content increased, and vice versa for the Ni 2p peak intensities. Furthermore, based on the depth profiles determined by XPS, there was no evidence of atomic diffusion between Pt and Ni, which indicated alloy formation in the film. Therefore, Ni-Pt alloy films deposited by the SFCD technique can be used as a suitable model for catalytic reactions due to their high activity and good stability for various reactions.

## 1. Introduction

Currently, the development of novel functional micro- and nanomaterials such as thin films [1,2], nanoparticles [3,4], nanowires [5,6], and nanorods [7,8] has attracted the interest of both scientists and industrial societies around the world. Such materials have been the subject of intense research in the field of inorganic or organic–inorganic hybrid materials aiming to improve the physicochemical properties compared to existing materials. In addition, the materials’ characteristics and novel application opportunities depend on the fabrication method, creating the need to design and optimize new synthetic approaches [9,10]. For these reasons, a variety of materials processing technologies are utilized in a wide range of applications areas, including microelectronic devices, optics, energy conversion/storage, chemistry, and catalysis. Among these, the use of supercritical fluids (SCF) could be considered as a versatile approach for designing micro and nano-materials due to their thermophysical properties. A SCF is a hybrid phase of both liquid and gas that is easily tunable with small variations in temperature or pressure. Under typical processing conditions, SCF are characterized by liquid-like density, gas-like viscosity, higher diffusivity than liquids, and zero surface tension [9,10,11,12,13,14,15,16,17,18,19,20].

The most frequently applied solvent in SCF is carbon dioxide, which is called supercritical carbon dioxide (scCO_2_). It has relatively mild critical temperature (*T_c_* = 31 °C) and critical pressure (*P_c_* = 7.38 MPa) conditions, which make CO_2_ a useful medium for a variety of applications, especially for thermochemical reactions. Moreover, by the addition of co-solvents (ethanol, acetone, hexane, isopropyl alcohol, etc.) in CO_2_, the solubility of polar precursors can be increased due to changes in polarity and density of the solvent, which makes it more attractive as a solvent medium for controlling the solubility of precursors in the processing of micro/nanomaterials [9,10]. One of the SCF-based technologies that has attracted interest in the development of functional materials is supercritical fluid chemical deposition (SFCD). This promising technique basically consists of the following process steps: Dissolution of the precursor in the SCF, adsorption of the precursor and a reducing agent onto the substrate surface, reduction of the adsorbed precursor to its metal form on the substrate surface by the reducing agent, and desorption of hydrogenated ligands from the substrate into the SCF phase [15,19].

The SFCD technique can be employed to deposit metals, metal oxides, alloys, and ceramic materials on crystalline substrates, supported materials, and polymers in the form of thin films or nanoparticles. Using this technique, many metals or metal oxides (Cu, Ni, Ru, Co, Pt, Pd, Au, Ag, etc.) have been successfully deposited onto a wide variety of substrates, with good conformal step coverage and high-aspect-ratio features [12,21,22,23,24,25,26,27]. However, for synthesized alloy films, the SFCD technique has been employed in relatively few studies, and only a few metal alloys have been reported such as Cu-Ag, Cu-Mn, and Cu-Ni. Zhao et al. reported that single-phase alloys (solid solutions) were not formed during deposition of Cu-Ag and Cu-Mn films, while Rasadujjaman et al. reported that the formation of a Cu-rich solid solution occurred after annealing the deposited films at a higher temperature [28,29,30]. Such alloy systems are typically known to involve complex intermetallic, phase-separated, and solid solution structures, which makes it difficult to obtain a better understanding of metal alloy deposition. For this reason, knowledge of alloy systems attainable by SFCD synthesis is still needed, especially study of the characteristics of metal alloy films.

Currently, one of the metal alloy films with the greatest development interest is Ni-Pt alloy thin films, which are widely used in many applications such as magnetic micro-electromechanical system (MEMS) devices [31], polymer electrolyte fuel cell (PEFC) cathodes [32], and especially for catalytic reactions as diverse as the oxygen reduction reaction (ORR) [32,33], hydrogen evolution reaction (HER) [34,35], and methanol/ethanol oxidation [9,36,37,38]. This is because Ni is a homologous element with Pt over a range of mass ratios and shows high catalytic activity and good stability for various reactions [31,32,39]. In addition, alloying Pt with Ni is expected to greatly reduce the overall cost by decreasing the use of noble metal in the prepared catalyst materials. Generally, the formation of a single-phase Ni-Pt alloy (solid solution) depends on the thermodynamics of the fluid system such as the phase diagram and the mixing enthalpy of the element pair. The Ni-Pt phase diagram exhibits full miscibility with the existence of ordered phases, and an approximately linear relationship of the variation Curie temperature (*T_C_)* on composition in the Ni-rich solid solution, yielding 100 °C at 27 at.% Pt [40]; whereas, the Ni-Pt system indicates a negative mixing enthalpy over the entire range of composition, favoring the formation of solid solution structures [41]. Moreover, the formation of single-phase Ni-Pt alloy that shows full miscibility thanks to their identical face-centered cubic lattice structure (fcc) and their equivalent atomic radii [42]. To date, Ni-Pt alloy thin films with varying compositions have been deposited by sputtering, electrodeposition, electron beam evaporation, and chemical vapor deposition (CVD) methods [9,31,32,33,34,43,44].

Herein, in this study, we demonstrate the feasibility of Ni-Pt alloy thin film deposition on TiN/SiO_2_/Si substrates through hydrogen reduction of nickel(II) hexafluoroacetylacetonate hydrate and platinum(II) hexafluoroacetylacetonate precursors from scCO_2_ solutions with various element compositions. In this work, we employed an SFCD flow-type reaction system, which is a single-step process enabling a simple integration procedure for alloy film deposition. Furthermore, we characterized the deposited Ni-Pt alloy thin films by using various analytical techniques.

## 2. Materials and Methods

### 2.1. Materials

The substrates used in this research were Si(100) wafers with a TiN/SiO_2_ layer on top. The other raw materials were liquid CO_2_, nickel(II) hexafluoroacetylacetonate hydrate [Ni(hfac)_2_**·**3H_2_O] and platinum(II) hexafluoroacetylacetonate [Pt(hfac)_2_] as precursors, H_2_ gas as the reducing agent, and acetone as co-solvent.

### 2.2. Synthesis of Ni-Pt Alloy Thin Films

**Figure 1:**
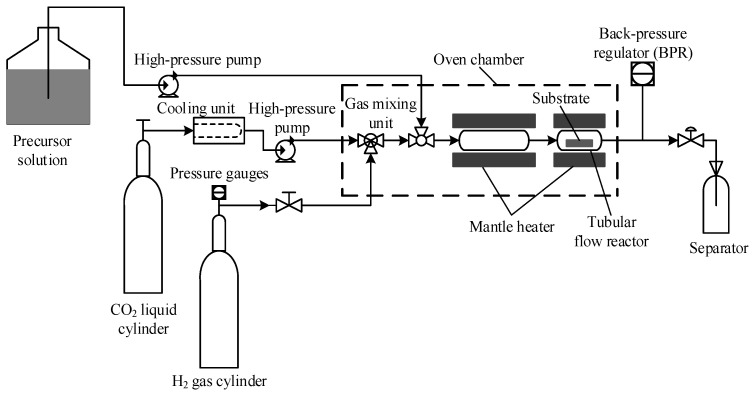
Schematic diagram of flow-type supercritical fluid chemical deposition (SFCD) reaction system.

In this study, Ni-Pt alloy thin films were prepared by a flow-type SFCD reaction system, whose detailed schematic diagram is shown in Figure 1 [12,17].

Ni(hfac)_2_·3H_2_O and Pt(hfac)_2_ precursors were mixed and stirred with acetone solvent listing these concentrations in Table 1 in a glass beaker, and hereafter this solution is referred to as the precursor solution. Meanwhile, the scCO_2_ solution was mixed with H_2_ gas by using a gas mixing unit. Then, the scCO_2_ solution (flow rate: 3.0 mL/min) was blended with the precursor solution (flow rate: 0.5 mL/min) by using a triangular pipe, and the mixture was continuously flowed into a tubular flow reactor. Before entering the reactor, the solution was preheated by the electric heater at 150 °C. Subsequently, the tubular flow reactor was heated by the electric heater (type: P-21, 100 V, 100 W) at 300–330 °C with a heating rate of 5 °C/min. All temperatures in the reactor were controlled by a type K calibrated thermocouple. Prior to the deposition process, the substrate was placed inside the tubular flow reactor, which has dimensions of 60 mm in length, 10 mm in inner diameter, and 1 mm in wall thickness. All of the equipment including the tubular flow reactor, gas mixing unit, and related valves and piping, were placed in an oven chamber maintained at 40 °C. The pressure of this system was controlled by a back-pressure regulator (BPR) at 10 MPa located downstream of the reactor. The deposition conditions of the Ni-Pt alloy thin films used in this work are summarized in Table 1. Furthermore, the thickness of the deposited Ni-Pt alloy thin films was measured using a Veeco-Dektak 150 profilometer.

### 2.3. Ni-Pt Alloy Thin Film Characterization

The deposited Ni-Pt alloy thin films were characterized in order to obtain their chemical composition, crystallinity, morphology, and topography. X-ray diffraction (XRD) analysis was carried out for crystallographic phase identification of the deposited Ni-Pt alloy thin films using a Shimadzu XRD-6000 (Shimadzu Corp., Kyoto, Japan) diffractometer with Cu Kα radiation at 40 kV and 30 mA in the 2θ range of 10–80°. Elemental analysis of the Ni-Pt alloy thin films was carried out by X-ray fluorescence with a Rigaku ZSX Primus wavelength dispersive X-ray fluorescence spectrometer. For surface morphology analysis, the Ni-Pt alloy thin film samples were observed by field-emission scanning electron microscopy coupled with energy-dispersive X-ray spectroscopy (FE SEM-EDX; JSM-6500, JEOL Ltd., Tokyo, Japan) at an acceleration voltage of 15 kV. Moreover, the chemical state of elements on the surface of the Ni-Pt alloy thin films and the depth profile were analyzed by X-ray photoelectron spectroscopy (XPS; JPS-9200, JEOL Ltd., Tokyo, Japan) with Mg Kα radiation operated at 15 kV and 10 mA accelerating voltage and emission current, respectively. Then, the Ni-Pt alloy thin film was observed by a scanning transmission electron microscope coupled with energy-dispersive X-ray spectroscopy (STEM-EDX; Tecnai Osiris, FEI, Eindhoven, Netherlands) with an acceleration voltage of 200 kV to gain an understanding of the crystalline topography and morphological structure.

## 3. Results and Discussion

### 3.1. Deposition of Ni-Pt Alloy Thin Films

Ni-Pt alloy thin films were synthesized by the SFCD technique from a precursor mixture of Ni(hfac)_2_**·**3H_2_O and Pt(hfac)_2_ with various elemental compositions via hydrogen reduction at different deposition temperatures, obtaining shiny, continuous, and reflective films. After the deposition of Ni-Pt alloy thin films, the film thickness was analyzed by Dektak-150 measurement (Table S1) and the deposition rates were calculated by dividing the total thickness by the deposition time.

**Figure 2:**
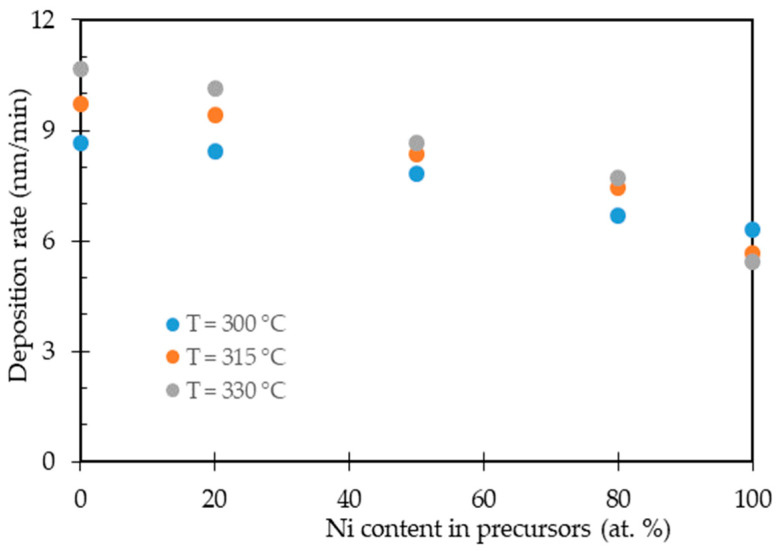
Deposition rate of Ni-Pt alloy thin films as a function of Ni content in the precursor solution.

Figure 2 shows the deposition rates for Ni-Pt alloy thin films with various elemental compositions as a function of temperature, which was varied over the temperature range 300 °C to 330 °C. At all temperatures, the deposition rate for Ni-Pt alloy thin films decreased with increasing Ni content from 20 to 80 (at.%) in the alloying systems. Decreasing the deposition rate of the Ni-Pt alloy thin films was due to the use of hydrate-based of Ni precursor, which this precursor species typically has low solubility in scCO_2_ owing to the presence of water molecules bound to Ni in the precursor Ni(hfac)_2_·3H_2_O [16]. Other authors have previously reported that the lower solubility values in scCO_2_ for hydrated precursor in the copper (II) hexafluoroacetylacetonate [45] and acetylacetonate complexes of cobalt and manganese [46]. In addition, increasing the deposition temperature increased the deposition rate of pure Pt films, whereas this phenomenon occurs in reverse for pure Ni films. Lee et al. reported that the highest growth rate for Pt thin films occurred in the temperature range 280 °C to 340 °C. At higher temperatures, the Pt precursor typically is more exothermic, which is enhanced the chemisorption between precursor and converting agent on surface-active sites of the substrate, resulting in a fast growth rate [47]. Battiston et al. reported that the starting temperatures for Pt film deposition should be at least 240 °C and they divided the deposition temperatures range 240–300 °C in the presence of oxygen and 280–440 °C in the water vapor. The Pt thin film deposition is an autocatalytic process in which Pt has an “induction period”; the time required to build up enough catalytic platinum to cause rapid acceleration of the deposition rate. The induction period for platinum can be achieved by increasing the temperature and the percentage of oxygen [48]. While Ni thin films have a tendency to show a decrease in growth rate at temperatures above 270 °C due to the thermal decomposition of Ni precursor, the resulting Ni precursor available for hydrogen reduction decreases in concentration with increasing reaction temperature above 270 °C [49]. For these reasons, the increase in the Ni content in the Ni-Pt alloy thin films caused a decrease in the deposition rate, which might be related to the grain/crystallite sizes of Ni and Pt elements on the surface of the substrate.

**Figure 3:**
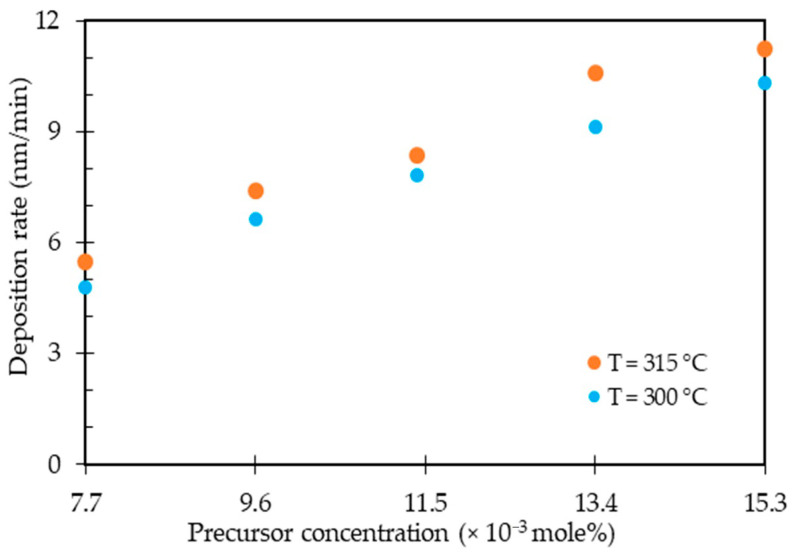
Deposition rate of Ni-Pt alloy thin films as a function of precursor concentration.

The effect of precursor concentration with the Ni/Pt ratio of 50:50 (at.%) on the deposition rate was observed at deposition temperatures of 300 °C and 315 °C, as shown in Figure 3. The deposition rate of Ni-Pt thin alloy films gradually increased as the precursor concentration was increased from 7.7 × 10^−3^ to 15.3 × 10^−3^ (mole%). Increasing the precursor concentration will lead to more collisions of that precursor, which may favor the precursor available for hydrogen chemisorption increases on surface-active sites of the substrate; this consequently increases the deposition rate of the Ni-Pt thin alloy films. In addition, increasing the precursor concentration results in more frequent molecular interactions between the molecules of precursors in a scCO_2_/acetone solvent that tend to form complexes such as hydrogen bond or van der Waals forces, which caused increasing the precursor solubility in the fluid system. Teoh et al. reported that the presence of inter- or intra-molecular hydrogen bonds in a scCO_2_/co-solvent can enhance the solubility of a number of polar solutes due to the increase of polarization of one of the C=O bonds of CO_2_ [50]. Regarding the temperature dependence, this figure also shows that the deposition rate increased with the increase in temperature. This is because the solubility of the precursors increases with temperature at higher concentrations, owing to the effect of solute vapor pressure being more dominant compared to the effect of a rapid decrease in density of the fluid, which yielded a higher growth rate for the Ni-Pt thin alloy films. The effect of temperature on solubility usually is more intricate due to the influence of both the density of the scCO_2_ solvent and the vapor pressure of the solute [16,50]. The highest deposition rate of Ni-Pt alloy thin films obtained was 11.25 nm/min at a precursor concentration of 15.3 × 10^−3^ (mole%) and a temperature of 315 °C.

To determine the exact elemental composition of the Ni-Pt alloy thin films, the final chemical composition of the deposited Ni-Pt films was measured by XRF as shown in the table below.

The relationship between the Ni-Pt ratio in the precursor solution and in the deposited film at temperatures of 300 °C, 315 °C, and 330 °C for different elemental compositions is tabulated in Table 2. The Ni-Pt ratio in the precursor solution did not agree with the final film composition. This may be related to differences in the crystallographic structure and atomic radii of the metals in the Ni-Pt alloy films [31,51].

**Figure 4:**
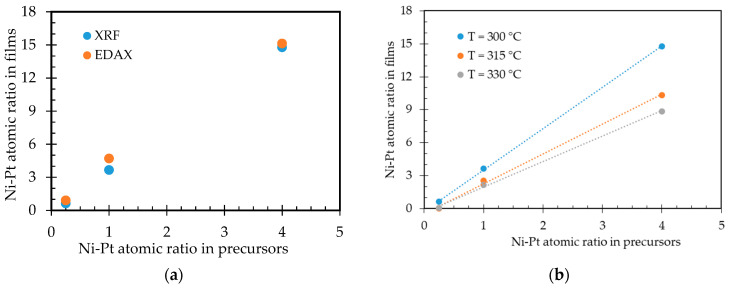
Ni-Pt atomic ratios in precursors and in films: (**a**) Determined by XRF and SEM-EDX; (**b**) at different deposition temperatures.

Furthermore, in this study, we analyzed the Ni-Pt atomic ratio in the precursor and in the film as determined by XRF and EDX measurements. From these results, the measurement of the Ni-Pt atomic ratio obtained by EDX was in fairly good agreement with that determined by XRF (see Figure 4a). In addition, Figure 4b shows the correlation between the Ni-Pt atomic ratio in the precursor solution used for deposition and that of the final films obtained at temperatures of 300 °C, 315 °C, and 330 °C. A good linear relation was observed between the Ni-Pt atomic ratio in the precursor solution and that in the films. However, there was a deviation in the values obtained from these correlations, which may be due to the different atomic radii of Ni and Pt [31].

### 3.2. Characterization of Ni-Pt Alloy Thin Films

The synthesized Ni-Pt alloy thin films were characterized by using various instrumental analysis techniques such as XRD, SEM-EDX, TEM-EDX, and XPS.

**Figure 5:**
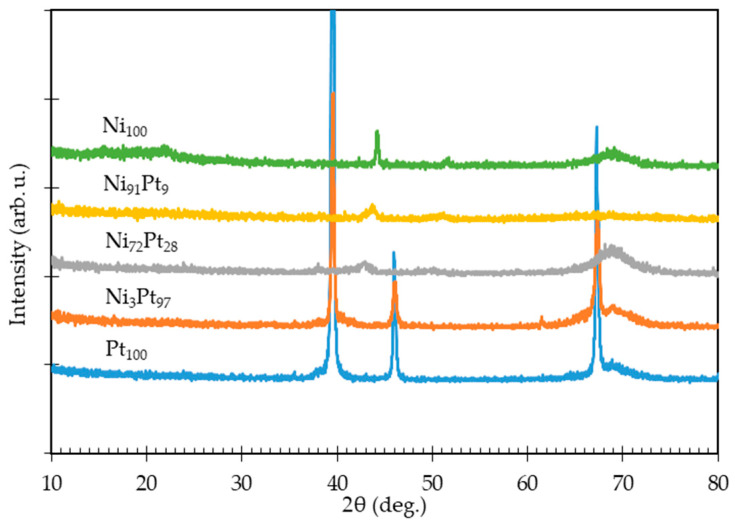
XRD patterns for deposited Ni-Pt alloy thin films at different elemental compositions.

The XRD patterns for Ni, Ni-Pt alloy, and Pt films with different elemental compositions deposited at 315 °C, 300 °C, and 330 °C are shown in Figure 5, Figure S1a,b respectively. The XRD spectra indicate that the yielded Ni and Pt films are purely in the metallic state, and no oxide peaks (Ni oxides and Pt oxides) are apparent. Sharp diffraction peaks for Ni thin films occurred at 2θ: 44.4° and 51.7° for Ni(111) and Ni(200), respectively (Ni JCPDS No. 040850), while for the Pt thin films, sharp diffraction peaks were observed at 2θ: 39.5°, 46.0°, and 67.2° for Pt(111), Pt(200), and Pt(220), respectively (Pt JCPDS No. 040802). Subsequently, the XRD peaks of the deposited Ni-Pt alloy thin films were located at angles between those of the Ni and Pt film peaks. These peaks shifted to lower diffraction angles with decreasing Ni content, which might indicate the solubility of nickel in the platinum lattice and a single-phase Ni-Pt alloy formation [43]. At all temperatures, the XRD patterns consistently exhibited the presence of a single-phase Ni-Pt alloy formation. Such shifted peaks of the Ni-Pt alloy films in this work agreed with the reports of Takahashi et al. [32], Eiler et al. [31,35], and Park et al. [38].

**Figure 6:**
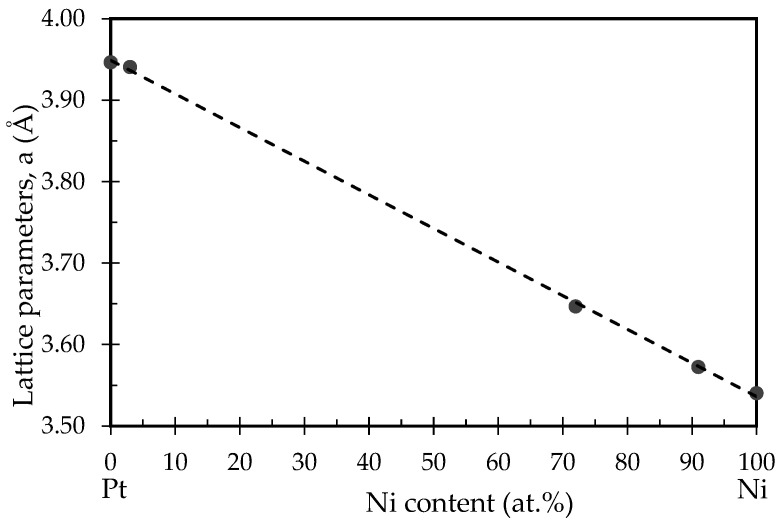
Lattice parameters as a function of Ni content in alloy films.

Figure 6 shows that the correlation between lattice parameters for face-centered cubic (fcc) Ni and fcc Pt with Ni content in the Ni-Pt alloy thin films followed Vegard’s Law. A plot of the cell parameter of the Ni-Pt phase against the alloy elemental composition showed good linearity, which is similar to results in the literature [31,32]. In addition, the calculated values of the lattice parameter (a) decrease with increasing Ni content (at.%) as determined in this work, as well as in the works of Takahashi et al., Eiler et al., and Zhang et al. [31,32,52]. According to Zhang et al., decreasing the Pt concentration in Ni-Pt alloys reduced the value of the lattice constant due to the smaller atomic radius of Ni than Pt [52]. Therefore, Ni-Pt alloys could be prepared in the Ni-Pt thin films for all elemental compositions.

**Figure 7:**
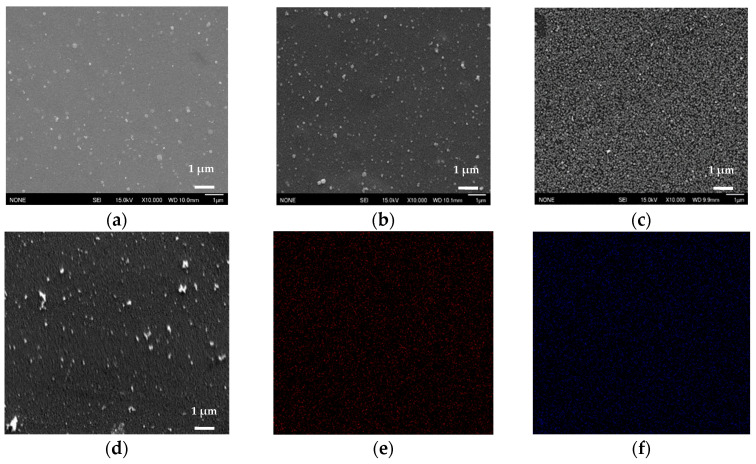
SEM images of deposited Ni-Pt alloy thin films: (**a**) Ni_91_Pt_9_; (**b**) Ni_72_Pt_28_; (**c**) Ni_3_Pt_97_; (**d**) EDX for Ni_72_Pt_28_ and corresponding elemental mapping images of (**e**) Ni; (**f**) Pt.

SEM was used to observe the surface morphology of the Ni-Pt alloy thin films. The top-surface SEM images of the Ni-Pt alloy thin films with different elemental compositions deposited at 315 °C and 300 °C are shown in Figure 7 and Figure S2, respectively. The deposited films are smooth, continuous, and uniformly distributed on the substrate surface for all elemental compositions studied, including Ni_91_Pt_9_, Ni_72_Pt_28_, and Ni_3_Pt_97_. The Pt surface in the Ni region was populated with dense and bright structures as shown in Figure 7a–c and the Ni-Pt alloy films exhibited an increase in appreciable microstructure with increasing Pt content, as shown for the Ni_3_Pt_97_ alloy film. In addition, a compositional map was created for the surface of the Ni_72_Pt_28_ alloy thin film by SEM-EDX (Figure 7d–f), which clearly showed a homogeneous distribution of Ni and Pt in the film.

**Figure 8:**
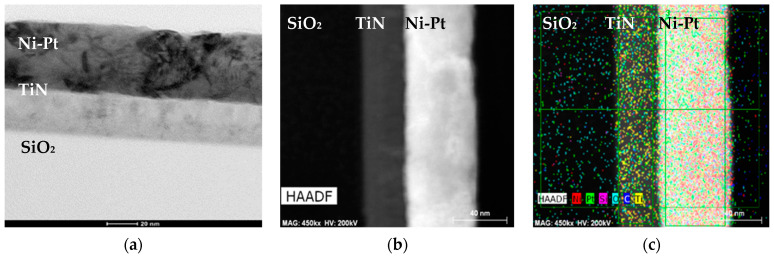
Cross-sectional (**a**) TEM; (**b**) -angle annular dark-field scanning transmission electron microscopy system (HAADF STEM); and (**c**) STEM EDX compositional map of deposited Ni_72_Pt_28_ alloy thin film.

The cross-sectional TEM image in Figure 8a illustrates the crystalline morphology of the Ni_72_Pt_28_ alloy thin film deposited by the SFCD technique at 315 °C. It is evident that the top surface of this Ni-Pt alloy thin films is polycrystalline and purely metallic. Furthermore, Figure 8b,c depicts the elemental distribution of Ni and Pt in the alloy films obtained by a high-angle annular dark-field scanning transmission electron microscopy system equipped with an energy-dispersive X-ray spectrometer (HAADF-STEM EDX). It can be clearly seen that a Ni-Pt alloy with the atomic ratio of 72:28 is uniformly distributed on the TiN/SiO_2_ substrate. However, a layer a few nanometers thick covers the top surface of the thin films, which may be a surface oxide or contamination layer caused by handling of the final product after the deposition process.

**Figure 9:**
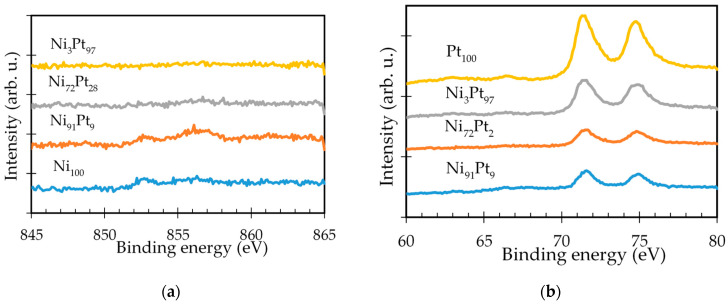
XPS spectra of deposited Ni-Pt alloy thin films with different elemental compositions: (**a**) Ni 2p; (**b**) Pt 4f.

To identify the chemical state of elements in the deposited Ni-Pt alloy thin films, XPS analysis was performed, as shown in Figure 9.

Figure 9a shows Ni 2p spectra of Ni-Pt alloy thin films with different elemental compositions deposited at 315 °C. In the Ni 2p spectra, the Ni_100_ and Ni_91_Pt_9_ films showed a Ni peak corresponding to metallic Ni at a binding energy of 852.9 eV [53]. However, the Ni_72_Pt_28_ and Ni_3_Pt_97_ films showed no Ni peaks at the film surface because of the dominance of Pt in the elemental distribution. For the Pt 4f spectra, the deposited Ni-Pt alloy thin films with different elemental compositions are shown in Figure 9b. Regarding the Pt 4f spectra, the binding energies of metallic Pt 4f7/2 and Pt 4f5/2 peaks were obtained at 71.5 eV and 74.8 eV, respectively. It can be clearly seen that the intensity of the Pt 4f peaks for the films decreased as the Ni content increased. In addition, the Pt 4f peaks are shifted to higher binding energy than that of the original peak position of the pure Pt sample, indicating either a change in the oxidation state of the element or a change in the electron configuration caused by the alloying of Pt with Ni [32,54].

**Figure 10:**
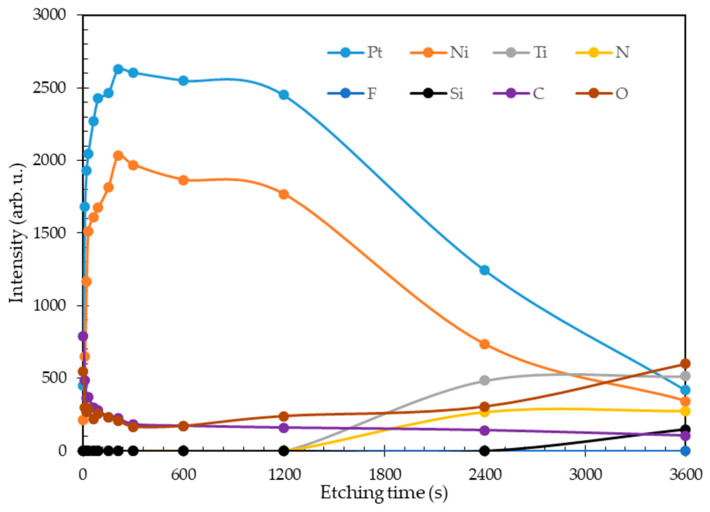
Depth profiles of Ni_72_Pt_28_ alloy thin films.

The XPS depth profile of the Ni-Pt alloy thin film with atomic ratio of 72:28 deposited at 315 °C is shown in Figure 10. As can be seen in this figure, the top surface is dominated by Pt and Ni, and the intensity of the peaks corresponding to these elements decreased with increasing etching time. In addition, all the Ni was distributed near the TiN film substrate, which indicated that Ni deposition can be initiated at a lower temperature than Pt, in agreement with our previous result concerning the effect of temperature on the deposition rate (see Figure 2). Moreover, no atomic diffusion occurred between Pt and Ni within the etching time range of 0 to 3600 s. Such a Ni-Pt depth profile indicated alloy formation between Ni and Pt due to full intermixing of Ni with Pt [38]. However, oxygen and carbon species are present on the Ni-Pt alloy thin films, indicating that the surface was covered by organics [35]. The presence of O and C signals may be due to acetone or organic by-products remaining on the film surface after the deposition process. Meanwhile, no F signal was observed, indicating that there was no contamination by the fluoro-based ligand complex. As the etching time reached 2400 s, titanium and nitrogen signals were detected, associated with the TiN film on the substrate used in this work.

## 4. Conclusions

Ni-Pt alloy thin films prepared by the SFCD technique from Ni(hfac)_2_**·**3H_2_O and Pt(hfac)_2_ precursors through hydrogen reduction in supercritical carbon dioxide solutions have been successfully synthesized and characterized. The deposition rate of the Ni-Pt alloy thin films decreased with increasing Ni content in the alloying systems, and gradually increased as the precursor concentration was increased to 15.3 × 10^−3^ (mole%). The film peaks shifted to lower diffraction angles with decreasing Ni content, as determined by XRD, indicating the presence of a single-phase Ni-Pt alloy formation. Obviously, with increasing deposition temperature from 300 °C to 330 °C (Figure S1c), a single-phase Ni-Pt alloy peaks became sharper, indicating that the crystallites were larger. The deposited films were smooth, continuous, and uniformly distributed on the substrate surface for all elemental compositions, as revealed by SEM-EDX and HAADF-STEM EDX analyses. In the XPS analysis, the intensity of the Pt 4f peaks of the films decreased as the Ni content was increased, and vice versa for the Ni 2p peak intensities. Furthermore, based on the depth profiles as determined by XPS, the top surface is dominated by Pt and Ni, and the intensity of peaks associated with those elements decreased with increasing etching time. In this profile, it was clear that no atomic diffusion occurred between Pt and Ni in the etching time range of 0 to 3600 s, indicating alloy formation in the film. Therefore, Ni-Pt alloy films deposited by the SFCD technique can be used as a suitable model for catalytic reactions such as ORR, HER, and methanol oxidation.

## Figures and Tables

**Table 1:** Deposition conditions for Ni-Pt alloy thin films.

Parameters	Value
Precursor concentration	(7.7–15.3) × 10^−3^ (mole%)
Deposition temperature	300–330 °C
H_2_ concentration	0.94 (mole%)
Total pressure	10 MPa
Deposition time	30 min
Flow rate of CO_2_ solution	3.1 mL/min
Flow rate of precursor solution	0.4 mL/min

**Table 2:** Elemental compositions of Ni-Pt in the precursors and in the deposited films.

Ni-Pt in the Precursors (at.%)	Ni-Pt in the Deposited Films (at.%)		
	T = 300 °C	T = 315 °C	T = 330 °C
Ni_100_	Ni_100_	Ni_100_	Ni_100_
Ni_80_Pt_20_	Ni_94_Pt_6_	Ni_91_Pt_9_	Ni_90_Pt_10_
Ni_50_Pt_50_	Ni_79_Pt_21_	Ni_72_Pt_28_	Ni_68_Pt_32_
Ni_20_Pt_80_	Ni_38_Pt_62_	Ni_3_Pt_97_	Ni_7_Pt_93_
Pt_100_	Pt_100_	Pt_100_	Pt_100_

## Data Availability

Data sharing not applicable.

## Supplementary Materials

The following are available online at https://www.mdpi.com/2079-4991/11/1/151/s1, Table S1: Thickness measurement for Ni-Pt alloy thin films, Figure S1: XRD patterns for deposited Ni-Pt alloy thin films at different elemental compositions: (a) T = 300 °C; (b) T = 330 °C; (c) Ni/Pt with precursor ratio of 50:50 (at.%) at different temperatures, Figure S2: SEM images of deposited Ni-Pt alloy thin films at temperature of 300 °C: (a) Ni_94_Pt_6_; (b) Ni_79_Pt_21_; (c) Ni_38_Pt_62_.
