# Supplementary material for: Synthesis and Characterization of Ni-Pt Alloy Thin Films Prepared by Supercritical Fluid Chemical Deposition Technique
Source: Nanomaterials (Basel). 2021 Jan 9;11(1):151. doi: 10.3390/nano11010151 (PMC7826905; doi:10.3390/nano11010151)
Supplement: Supplementary file 1 [file nanomaterials-11-00151-s001.pdf]

Article

Synthesis and Characterization of Ni-Pt Alloy Thin Films Prepared by Supercritical Fluid Chemical Deposition Technique

Sudiyarmanto [1,2], and Eiichi Kondoh [1,*]

[1] Integrated Graduate School of Medicine, Engineering, and Agricultural Sciences, University of Yamanashi, Kofu, 400-8511, Japan; g18dtea3@yamanashi.ac.jp
[2] Research Center for Chemistry, Indonesian Institute of Sciences (LIPI), Kawasan PUSPIPTEK Serpong, Tangerang Selatan, Banten, 15310, Indonesia; sudi012@lipi.go.id
* Correspondence: kondoh@yamanashi.ac.jp; Tel.: +81-55-220-8472

1. Thickness measurement for Ni-Pt alloy thin films

Table S1. Thickness measurement for Ni-Pt alloy thin films

Sample name	Thickness (nm)		
	T = 300 °C	T = 315 °C	T = 330 °C
Pt_{100}	259.26	291.66	320.27
$Ni_{20}Pt_{80}$	252.60	282.60	304.25
$Ni_{50}Pt_{50}$	234.28	250.55	259.26
$Ni_{80}Pt_{20}$	200.64	223.90	231.38
Ni_{100}	188.71	169.50	162.66

2. XRD patterns

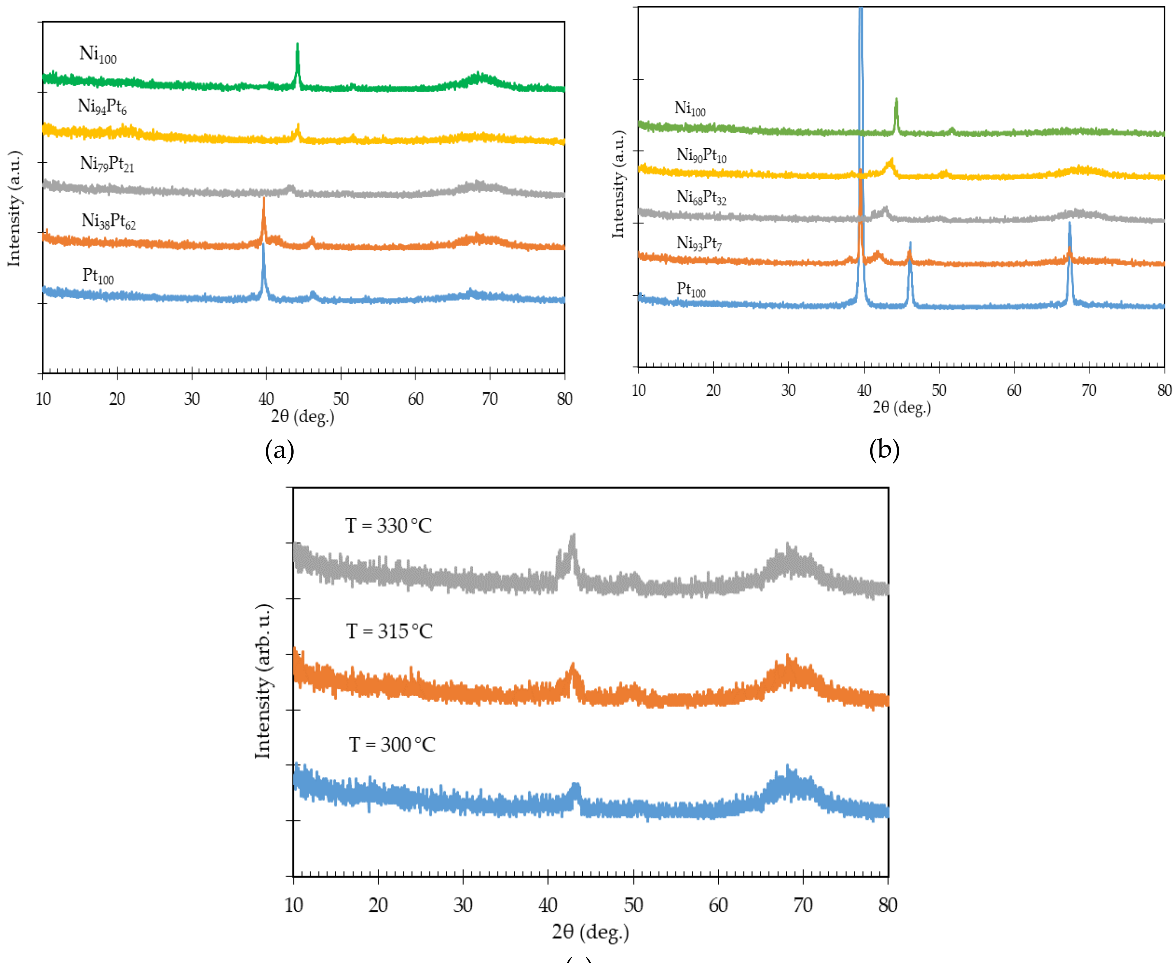

Figure S1. XRD patterns for deposited Ni-Pt alloy thin films at different elemental compositions: (**a**) T = 300 °C; (**b**) T = 330 °C; (**c**) Ni/Pt with precursor ratio of 50:50 (at.%) at different temperatures

3. SEM images

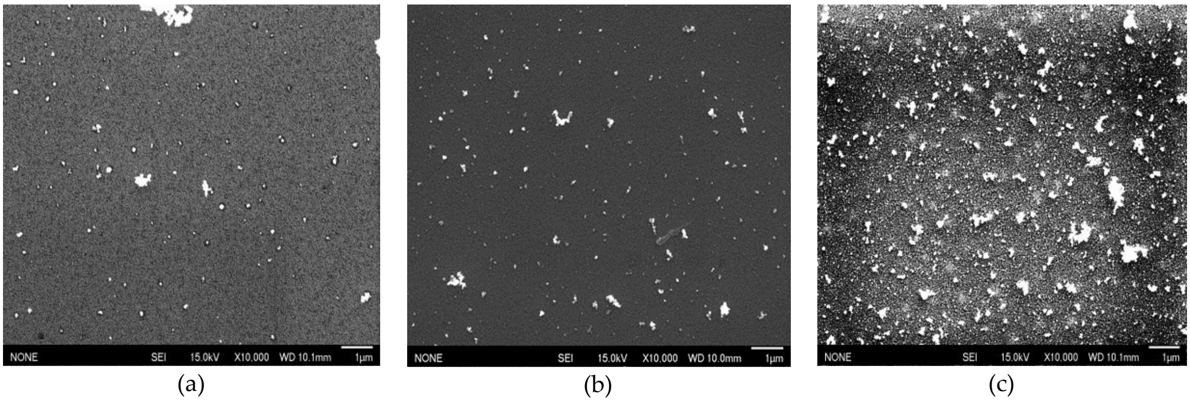

Figure S2. SEM images of deposited Ni-Pt alloy thin films at temperature of 300 °C: (**a**) $Ni_{94}Pt_6$; (**b**) $Ni_{79}Pt_{21}$; (**c**) $Ni_{38}Pt_{62}$
